# Test–Retest Reliability of a Conventional Gait Model for Registering Joint Angles during Initial Contact and Toe-Off in Healthy Subjects

**DOI:** 10.3390/ijerph18031343

**Published:** 2021-02-02

**Authors:** Francisco Molina-Rueda, Pilar Fernández-González, Alicia Cuesta-Gómez, Aikaterini Koutsou, María Carratalá-Tejada, Juan Carlos Miangolarra-Page

**Affiliations:** 1 Motion Analysis, Ergonomics, Biomechanics and Motor Control Laboratory (LAMBECOM), Department of Physical Therapy, Occupational Therapy, Rehabilitation and Physical Medicine, Faculty of Health Sciences, Rey Juan Carlos University, 28922 Madrid, Spain; francisco.molina@urjc.es (F.M.-R.); alicia.cuesta@urjc.es (A.C.-G.); maria.carratala@urjc.es (M.C.-T.); juan.miangolarra@urjc.es (J.C.M.-P.); 2 International Doctorate School, Rey Juan Carlos University, 28933 Madrid, Spain; 3 Department of Information Systems Engineering, University San Pablo CEU, Boadilla del Monte, 28668 Madrid, Spain; aikaterini.koutsou@ceu.es; 4 Physical Medicine and Rehabilitation Service of the University Hospital of Fuenlabrada, 28942 Madrid, Spain

**Keywords:** biomechanics, gait analysis, movement assessment, optical motion capture, reliability

## Abstract

The aim of this study was to evaluate the test–retest reliability of a conventional gait model (CGM), the Plug-in Gait model, to calculate the angles of the hip, knee, and ankle during initial contact (IC) and toe-off (TO). Gait analysis was performed using the Vicon Motion System^®^ (Oxford Metrics, Oxford, UK). The study group consisted of 50 healthy subjects. To evaluate the test–retest reliability, the intraclass correlation coefficient (ICC), the standard error of measurement (SEM), the minimal detectable change (MDC), and the Bland–Altman analysis with 95% limits of agreement were calculated. The ICC for the joint angles of the hip, knee, and ankle was higher than 0.80. However, the ankle angle at IC had an ICC lower than 0.80. The SEM was <5° for all parameters. The MDC was large (>5°) for the hip angle at IC. The Bland–Altman analysis indicated that the magnitude of divergence was between ±5° and ±9° at IC and around ±7° at TO. In conclusion, the ICC for the plug-in gait model was good for the hip, knee, and ankle angles during IC and TO. The plots revealed a disagreement between measurements that should be considered in patients’ clinical assessments.

## 1. Introduction

Three-dimensional gait analysis (3DGA) has an important role in the evaluation of gait disturbances [1]. The 3DGA motion capture systems have been considered the gold standard in quantitative gait analysis [2]. Despite its high accuracy and reliability, 3DGA remains underused in clinical settings due to high costs, difficulty in the interpretation of captured data, and technical requirements that this technology implies [3].

The 3DGA is often used to assess kinematics to discriminate gait patterns and examine change over time [4]. Most of the objective research in functional evaluation and biomechanics is mainly based on the use of optoelectronic systems [5]. One of the most common 3DGA analysis models implemented is the conventional gait model (CGM), which refers to a group of similar models with different names, such as Plug-in Gait, Vicon Clinical Manager, Newington, and Helen Hayes [6,7,8]. In these models, kinematic outputs are mainly joint angles describing the orientation of the distal segment with respect to that of the proximal segment (except the orientation of the pelvis) [9]. The most used alternatives to the CGM are the six-degree-of-freedom (6DoF) models. They track the segments independently (without constraining the joints) and can be based on rigid marker clusters [9].

Most reliability studies have analyzed the range of motion and the peak angles during walking [4,10,11,12,13,14,15]. However, they have not studied the test–retest reliability of gait models to obtain the joint angles during specific phases of the gait cycle, such as the initial contact or the toe-off. In this sense, gait rehabilitation after a neurological disorder frequently focuses on achieving the adequate joint pattern in specific gait phases, such as the hip flexion in the initial contact phase or the plantar flexion in pre-swing [16,17,18]. In addition, most gait observation scales used in clinical settings focus their analysis on joint angles in specific phases of the gait [19].

The reliability of kinematic measurements has an important priority in gait analysis [12]. This is necessary to detect significant changes after clinical practice or research interventions [20]. Variability in 3D gait analysis is mainly due to intrinsic and extrinsic variations. Intrinsic variations reflect intra-individual oscillations that arise naturally, through either trial-to-trial or subject-to-subject variability [21,22,23]. Extrinsic variations correspond to measurement error and can be due to different causes, such as marker placement, skin motion, data processing, and assessors’ experience [13]. Consequently, it is important to identify the measurement error for these outcomes to avoid misinterpretation of the results. Knowledge about test–rest reliability, minimal detectable change (MDC) values, and the limits of agreement from healthy population is very important since it can help clinicians and researchers interpreting pathological data [14]. Therefore, the aim of the present research was to evaluate the test–retest reliability of a CGM, the Plug-in Gait model, to obtain the hip, knee, and ankle angles at initial contact (IC) and toe-off (TO) during walking.

## 2. Materials and Methods

### 2.1. Participants

The sample consisted of a total of 50 subjects, 24 males and 26 females. The ages of the patients ranged from 20 to 34 years (mean age of 21.62 ± 2.62 years). The mean body mass was 65.74 ± 12.94 kg, and the mean height was 167.49 ± 25.57 cm. This sample participated in a previous protocol [24].

Recruitment was based on deliberate participation of subjects through informative meetings. Individuals were eligible if they met the following inclusion criteria: age ≥ 18 years, absence of pathologies that affect gait and/or posture, and walking without assistive products or technical aids.

The protocol was approved by the local ethical committee (0702201703417) of the Rey Juan Carlos University. Informed consent was obtained from all participants included in this study.

### 2.2. Procedure

The research was carried out at the Motion Analysis, Biomechanics, Ergonomics, and Motor Control Laboratory (LAMBECOM), located at the Faculty of Health Sciences, Rey Juan Carlos University (Madrid, Spain).

For gait analysis, the Vicon Motion System^®^ (Oxford Metrics, Oxford, UK) was used. This system is a three-dimensional motion analysis system composed of eight 100 Hz infrared cameras, three force plates (AMTI^®^, Watertown, NY, USA), and two video cameras (BASLER A601FC-2, Exton, PA, USA).

All subjects were measured twice with a separation of 1 week between meetings, and five repetitions were recorded per session. To perform the motion capture, reflective markers were attached to specific anatomical areas of the lower limbs according to the plug-in gait market set [25]. After the instrumentation was completed, the subjects walked along an 11 m walkway (back and forth) at a comfortable speed.

### 2.3. Analysis of Data

Five gait cycles per session for each subject were analyzed. The angles of the hip, knee, and ankle joints in the sagittal plane were analyzed at IC and TO during walking. Foot contact events were identified using a 20 N threshold on the vertical force component obtained by the platforms [24]. The Vicon Nexus software v1.8.5 was used to calculate outcome measures based on the biomechanical model of the Plug-in Gait model. The output angles for all joints were calculated from the YXZ Cardan angles derived by comparing the relative orientations of the two segments [25]. Through Vicon Polygon (Oxford Metrics Group, Oxford, UK), kinematic data were extracted to Microsoft Excel files.

### 2.4. Sample Size Calculation

The sample size was calculated based on the research of Walter et al. [26]. We calculated the sample size using the intraclass correlation coefficient (ICC) and the number of observers (*n* = 2). Considering a minimally acceptable intraclass correlation coefficient (ICC) (p0) of 0.60 and an expected ICC (p1) of 0.80, and following the contingency table of Walter et al., the sample size needed was 39 subjects. Considering a possible 10% attrition, 43 participants were required. Finally, the sample consisted of 50 participants.

### 2.5. Statistical Analysis

Statistical analysis was performed using the SPSS statistical software system (SPSS Inc., Chicago, IL; version 22.0). To calculate the reliability between the sessions, the ICC (absolute agreement and a mixed-effects model, ICC 3.1) and the 95% confident intervals were calculated [27]. The ICC values are interpreted as excellent (>0.90), good (0.76–0.90), moderate (0.50–0.75), and poor (lower than 0.50) [28].

Absolute reliability was defined by estimating the standard error of measurement (SEM), the minimal detectable change (MDC), and the standard deviation of the differences between raters (SDdiff). The SEM and MDC were determined using the following calculations: SEM = SDdiff × √1-ICC and MDC95 = 1.96 × √2 × SEM.

Bland–Altman plots with 95% limits of agreement were also obtained. The mean score and the difference between measurements (and the mean of the differences ±1.96 SD, standard deviation) were analyzed. The width of the limits of agreement and the distance of the mean of the differences between measurements with respect to zero can be used to explain the errors between assessments. These plots allow similarities between two different measurements when assessing the same dataset to examine the match level [29].

## 3. Results

In the study, 50 subjects participated, and there were no missing data. The gait velocity of the participants was 1.22 ± 0.11 m/s in the first measurement and 1.23 ± 0.12 m/s in the second measurement.

The reliability of kinematic parameters in the sagittal plane (joint angles of the hip, knee, and ankle at IC and TO) was examined. Results are shown in Table 1. The joint angles at TO of the hip, knee, and ankle had ICC greater than 0.80. The hip and knee angles at IC also had ICC greater than 0.80. However, the ankle angle at IC had an ICC lower than 0.80. The SEM was <5° for all the parameters. The MDCs were large (>5°) for the hip angle at IC.

**Figure 1:**
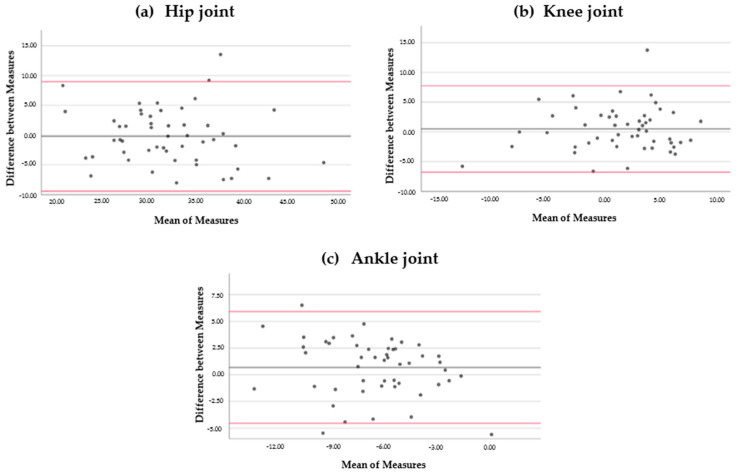
Bland–Altman plots. Difference between measurements for hip angles (**a**), knee angles (**b**), and ankle angles (**c**) during initial contact. Bias (black line) and limits of agreement (red lines) are indicated. The mean score is shown on the *x*-axis, and the difference between measures (and the mean of the differences) is shown on the *y*-axis (mean difference ± 1.96 SD).

**Figure 2:**
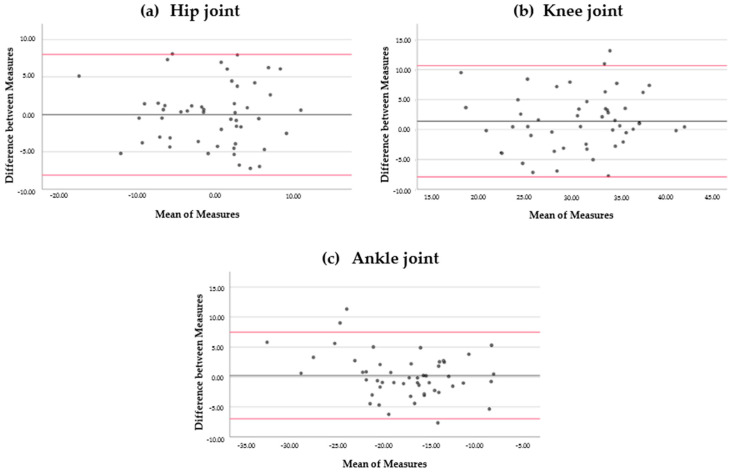
Bland–Altman plots. Difference between measurements for hip angles (**a**), knee angles (**b**), and ankle angles (**c**) during toe-off. Bias (black line) and limits of agreement (red lines) are indicated. The mean score is shown on the *x*-axis, and the difference between measures (and the mean of the differences) is shown on the *y*-axis (mean difference ± 1.96 SD).

The means of the measurements for the hip, knee, and ankle angles were 32.03°, −0.66°; 1.49°, 30.75°, and −6.58°, −17.80°, at IC and TO, respectively. In the Bland–Altman analysis, the limits of agreement for the hip, knee, and ankle angles were 8.99° to −9.39°, 7.99° to −8.11°; 7.74° to −6.78°, 10.68° to −7.94°, and 5.93° to −4.54°, 7.46° to −6.98°, at IC (Figure 1) and TO (Figure 2), respectively (Table 1).

## 4. Discussion

The purpose of the present study was to evaluate the test–retest reliability of a CGM (Plug-in Gait) to analyze the joint angles during the initial contact and toe-off phases of walking.

This study found that the Plug-in Gait is a reliable tool for obtaining the joint angles during specific phases of gait. The results obtained showed a good test–retest reliability (ICC > 0.80) for the joint angles at TO of the hip, knee, and ankle. At IC, the reliability was good for the hip and knee angles (ICC > 0.80) and moderate for the ankle angle (ICC < 0.80).

Most studies about gait models for 3DGA have analyzed ranges of motion or maximum joint peaks [14,30,31].

Previous studies have shown that the ICC for 3DGA is typically higher than 0.80 for kinematic data in the sagittal plane [15]. In this study, the ICC was reasonable for joint angles at IC and TO. Nevertheless, evaluation using ICC provides information regarding the relative reliability of measurements but can be of limited help when determining whether an observed change is due to an actual change in performance [32]. In this sense, the Bland–Altman analysis and MDC may be more valuable than the ICC as they can be readily and easily interpreted in a significant way in both the research and clinical context. Specifically, the width of the limits of agreement is helpful to know the level of agreement or disagreement between assessments [30,33]. In addition, MDC provides a meaningful and practical assessment of measurement error by providing a single value for each variable in the units of measure [31,32].

In our study, the Bland–Altman analysis indicated that the amount of disagreement was between ±5° and ±9° at IC and around ±7° at TO. These results are similar to the angles obtained in previous studies. Meldrum et al. noticed an amplitude of ±8° to distinguish the angle of the ankle during initial contact. The authors did not analyze the joint angle of the hip and knee during specific events of the gait cycle, but they studied the test–retest reliability for the range of motion and the peak angles, obtaining better agreement for these parameters (±4° for amplitude and about ±6° to 8° for peak kinematics in the sagittal plane) [30]. As for the measurement errors, McGinley et al. [15] determined that an error of less than 2° for a 3DGA system is considered appropriate in a clinical context, errors of between 2° and 5° are also rational but may involve caution in data explanation, and errors of more than 5° should raise concern and may be large enough to mislead clinical evaluation. The agreement obtained in this study for Plug-in Gait is not enough to detect minor variations between assessments. Disagreements in joint angles during IC and TO of less than 9° after an intervention could be due to system or observer error. In this sense, there are several sources of inconsistency, such as marker placement error, processing errors, and marker position errors [34].

Concerning MDC values, hip and knee kinematic angles at initial contact show the greatest MDC values (5.24 for the hip position and 4.29 for the knee position). The MDC values presented in this study suggest that caution should be used when interpreting small within-subject or between-group differences in joint angles at IC and TO. Fortunately, for many studies currently published, MDC values are commonly above the MDC values obtained in this study in healthy individuals [31]. Wilken et al. obtained MDC values of between 2.5° and 6° for hip parameters, between 4° and 7° for knee parameters, and around 3° for ankle parameters [31]. Meldrum et al. showed MDC values of around 4° and 8° for the hip joint, around 6° and 7° for the knee joint, and between 8° and 10° for the ankle joint [30]. Finally, Fernandez et al. registered MDC values of around 8° for the hip joint, about 4° for the knee joint, and about 4° for the ankle joint [24].

Despite our findings, some limitations of our study should be noted. For instance, the results on reliability cannot be generalized for the majority of gait phases. Therefore, a similar protocol could be used to analyze the test–retest reliability of the Plug-in Gait model to obtain the joint angles in specific gait phases, such as the loading response or the swing period. In addition, we consider that it is necessary that further studies analyze the test–retest reliability of the most important kinematic events in the frontal and transverse planes (hip adduction and abduction, hip external rotation, knee valgus, and ankle pronation). These events should be evaluated in the gait phases when these events occur (loading response and swing period). Additionally, this study does not use the CGM update. Currently, there is a new version, the CGM2 [35]. However, a validation study is still required to compare this model with the CGM1.

## 5. Conclusions

The intraclass correlation coefficient of the Plug-in Gait model was good (0.76–0.90) for the joint angles of the hip, knee, and ankle during the initial contact and toe-off phases of walking for the agreement between measurements. Despite this, the Bland–Altman analysis revealed a disagreement between measurements that should be evaluated in the analysis of the clinical assessments (between ±5° and ±9° at initial contact and around ±7° at toe-off).

## Figures and Tables

**Table 1:** Test–retest reliability for kinematic parameters.

Parameter	ICC	(95% CI)	Mean	D	SD (DIFF)	95% LOA	SEM	MDC
Hip angle at IC (°)	0.837	0.712–0.907	32.03	0.2	4.69	−9.39 → 8.99	1.98	5.24
Knee angle at IC (°)	0.825	0.693–0.901	1.49	0.47	3.70	−6.78 → 7.74	1.55	4.29
Ankle angle at IC (°)	0.774	0.602–0.871	−6.58	0.69	2.67	−4.54 → 5.93	1.27	3.52
Hip angle at TO (°)	0.880	0.788–0.932	−0.66	0.05	4.10	−8.11 → 7.99	1.03	2.85
Knee angle at TO (°)	0.814	0.671–0.895	30.75	1.37	4.75	−7.94 → 10.68	0.96	3.30
Ankle angle at TO (°)	0.880	0.788–0.9321	−17.80	0.24	3.68	−6.98 → 7.46	1.077	2.56

ICC, intraclass correlation coefficient; 95% CI, 95% confidence interval for the ICC; mean, mean of measurements at time 1 and time 2; D, mean of the differences between measurements at time 1 and time 2 and its standard deviation SD(DIFF); 95% LOA, Bland and Altman 95% limits of agreement. SEM, standard error of measurement; MDC, minimal detectable change.

## Abbreviations

The following abbreviations are used in this manuscript:

CGM	conventional gait model
LAMBECOM	Laboratory of Analysis of Movement, Biomechanics, Ergonomics, and Motor Control
IC	initial contact
TO	toe-off
ICC	intraclass correlation coefficient
CI	confidence interval
SD	standard deviation
SEM	standard error of measurement
MDC	minimal detectable change
LOA	limits of agreement
